# Prakriti phenotyping in Central Serous Retinopathy (CSR): case series on Ayurvedic therapeutic outcomes in Vata individuals

**DOI:** 10.3389/fmed.2025.1722235

**Published:** 2025-12-18

**Authors:** Shanti. K, Gopinathan. G

**Affiliations:** 1 Amrita Vishwa Vidyapeetham Amrita School of Ayurveda, Kollam, India; 2 Govt Ayurveda Hospital, Kannur, India

**Keywords:** Ayurveda, personalized medicine, CSCR, Shopha, macular edema

## Abstract

**Background:**

Central Serous Retinopathy (CSR) is a retinal disorder characterized by serous detachment of the neurosensory retina, commonly associated with psychological stress and Type A personality traits. From an Ayurvedic perspective, these features correspond to Vata predominance, marked by hyperactivity, anxiety, and autonomic instability. Such individuals may be constitutionally predisposed to disorders of Vata imbalance, including retinal pathologies resembling *Vataja Shopha*. This case series explores therapeutic outcomes of Ayurvedic management in CSR among individuals with *Vata Prakriti*.

**Case presentation:**

Three patients-two males (aged 65 and 46 years) and one female (aged 40 years), diagnosed with CSR and confirmed as *Vata Prakriti* through a standardized questionnaire, were treated with an identical Ayurvedic regimen comprising *Ashwagandha* tablets, *Punarnavadi Kashaya, Tiktaka Ghrita, Kalyanaka Ghrita*, and *Triphala Churna*. Local ocular therapies included *Shiro Pichu* with *Brahmi Ghrita, Pratimarsha Nasya* with *Anu Taila*, and *Anjana Karma* with *Elaneer Kozhumbu*. Lifestyle advice emphasized regular sleep, timely meals, guided meditation, and reduced screen exposure. All three patients demonstrated marked improvement in visual acuity and complete anatomical resolution of macular fluid on Optical Coherence Tomography within 4–17 months. No recurrence was observed over 1 year of follow-up.

**Conclusion:**

This case series highlights a possible association between CSR and *Vata Prakriti*. Vata pacifying and *Rasayana* based Ayurvedic interventions showed favorable visual and structural outcomes. Incorporating *Prakriti* phenotyping in ocular disease management may enhance personalized, integrative approaches to retinal disorders.

## 1 Introduction

Central Serous Retinopathy (CSR) is a retinal disorder characterized by the accumulation of subretinal fluid at the macula, leading to blurred or distorted central vision and increased macular thickness on optical coherence tomography (OCT) (1). The condition primarily affects young to middle-aged adults and has gained significance due to its potential to cause lasting visual deficits in a working-age population.

Psychological stress and certain behavioral traits have been consistently implicated in the onset of CSR. Individuals exhibiting Type A personality characteristics such as impatience, competitiveness, and emotional overreactivity show a higher prevalence of CSR compared with those without these traits (2, 3). Recent studies also report elevated anxiety and depression scores among CSR patients compared to controls with other ocular disorders. This psychobehavioural pattern is associated with heightened sympathetic activity, a physiological feature that appears central to CSR pathophysiology.

Autonomic imbalance, particularly sympathetic predominance with reduced parasympathetic control, has been demonstrated in CSR (4). Tewari et al. reported increased baseline sympathetic tone and reduced parasympathetic activity in CSR patients compared with healthy individuals (5). Such overactivation of the sympathetic system during stress may induce choroidal vascular hyperperfusion, compromise retinal pigment epithelial integrity, and subsequently cause neurosensory detachment (6). These mechanisms suggest that CSR represents not only a retinal vascular dysfunction but also a systemic neurohumoral imbalance driven by stress reactivity.

In Ayurvedic medicine, the concept of Prakriti describes an individual's constitutional type determined by the predominance of the three doshas-Vata, Pitta, and Kapha. Among them, Vata governs movement, neural regulation, and sensory coordination. Individuals with Vata predominant Prakriti tend to exhibit traits such as mental restlessness, irregular sleep, emotional sensitivity, and heightened responsiveness to external stimuli. These constitutional features bear close resemblance to the Type A behavioral pattern and to physiological states characterized by sympathetic overactivity. Vata aggravation, described as a state of instability and depletion, may thus parallel the autonomic hyperresponsiveness implicated in CSR.

Despite substantial advances in diagnostic imaging and interventional options such as photodynamic therapy, focal laser treatment, and pharmacological modulation, current CSR management predominantly focuses on anatomical correction (7). Little emphasis is placed on individual constitutional predisposition or stress reactivity, leaving a gap in personalized treatment approaches.

In this context, integrating Prakriti-based assessment into CSR management may help identify vulnerable individuals, optimize therapeutic regimens, and reduce recurrence. The present case series describes three CSR patients, all confirmed to have Vata-predominant Prakriti using a validated questionnaire, who showed complete anatomical and functional recovery following a standardized Ayurvedic regimen. By presenting consistent outcomes in constitutionally similar individuals, this series highlights the potential of Prakriti phenotyping as an adjunct framework for individualized care in ophthalmology. It aligns with the growing global emphasis on precision medicine, suggesting that integrating traditional constitutional models may enhance understanding and management of retinal disorders such as CSR.

## 2 Case presentation

### 2.1 Case 1

A 65-year-old non-diabetic, non-hypertensive male presented with blurred vision in his left eye of 2 weeks' duration. His visual acuity was 6/6 in the right eye and 6/36 in the left eye. Fundus examination revealed serous detachment of the neurosensory retina at the macula, confirmed on OCT by subretinal fluid accumulation with a central macular thickness (CMT) of 488 μm.

His Prakriti, assessed using the standardized CCRAS Prakriti Assessment Questionnaire, was Vata-predominant, characterized by a lean body, dry skin, and irregular sleep. Recent psychological stress due to family issues and irregular dietary habits were noted as *Vata-provoking* factors.

He underwent Ayurvedic treatment as detailed below, leading to gradual improvement of visual acuity to 6/9 and complete resolution of macular fluid on OCT after 4 months. No recurrence was observed during 6-month and 1-year follow-up.

### 2.2 Case 2

A 40-year-old female presented with blurred vision in the left eye of 1 week's duration. Visual acuity was 6/6 (right) and 6/60 (left). Fundus examination revealed fluid accumulation at the macula with soft drusen. OCT showed disruption of the retinal pigment epithelium (RPE) and subretinal fluid with CMT 457 μm.

Her Prakriti was assessed as Vata-predominant by the same standardized tool. She reported high occupational stress and irregular meal timings, consistent with *Vata aggravation*.

Following Ayurvedic management, progressive visual improvement was recorded, and OCT at 6 months demonstrated complete resolution of macular edema. No recurrence was noted at 6-month and 1-year follow-up visits.

### 2.3 Case 3

A 46-year-old non-diabetic, non-hypertensive male complained of blurred vision predominantly in the left eye. His visual acuity was 6/6 (right) and 6/12 (left). Fundus examination revealed macular edema, and OCT confirmed subretinal fluid accumulation with CMT 339 μm.

Prakriti assessment revealed Vata predominance, with constitutional features of mental restlessness, irregular sleep, and dry skin and constipation.

He received the same Ayurvedic regimen for 1 year and 5 months, after which macular edema resolved completely on OCT. Long-term follow-up showed no recurrence of the condition.

### 2.4 Therapeutic intervention (common to all cases)

All three patients were treated with a uniform Ayurvedic protocol aimed at *Vata shamana* (pacification of Vata Dosha) and *Rasayana chikitsa* (rejuvenation of ocular tissues).

The regimen comprised:

- Internal medications: *Tab. Ashwagandha, Punarnavadi Kashaya, Tiktaka Ghrita, Kalyanaka Ghrita*, and *Triphala Churna*.
- External/local therapies: *Siro Pichu* with *Brahmi Ghrita, Pratimarśa Nasya* with *Anu Taila*, and *Añjana Karma* with *Elaneer Kozhumbu*.
- Lifestyle and dietary advice: Regular sleep, timely meals, avoidance of fasting, stress, and excessive screen exposure-measures recommended for *Vata pacification*.

**Figure 1:**
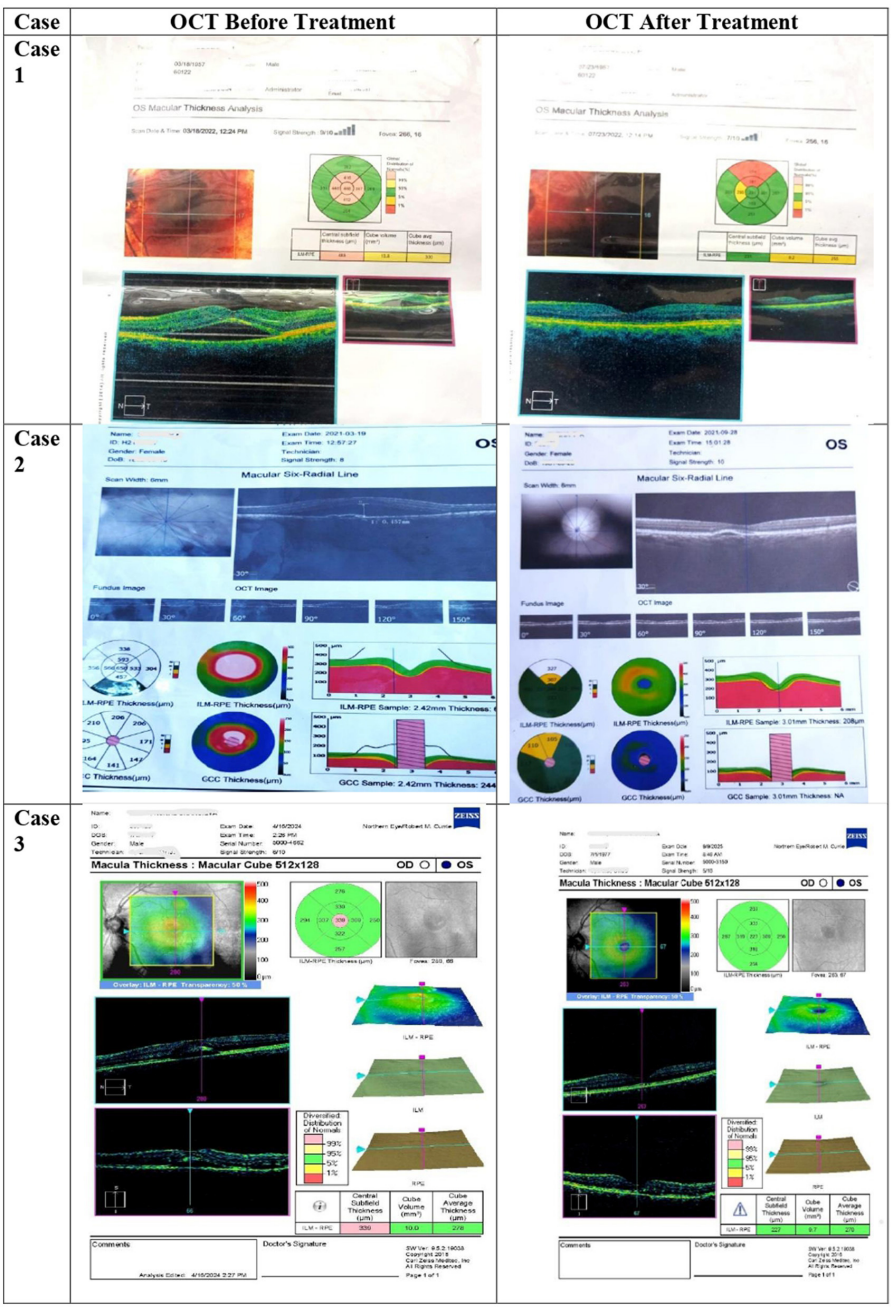
OCTs taken before and after the treatment.

The treatment duration was not pre-determined, instead it was guided by documented reduction in sub-retinal fluid on OCT, improvement in visual acuity and clinical assessment of symptom relief. Treatment duration ranged from 4 months to 15 months, depending on clinical progress. Table 1 shows a summary of therapeutic interventions. All patients exhibited gradual visual recovery, complete resolution of macular fluid on OCT, and absence of recurrence over at least 1 year of follow-up. Table 2 shows a summary of outcomes. Figure 1 shows the OCTs of the patients before and after the treatment.

**Table 1:** Summary of therapeutic interventions.

**Component**	**Formulation/procedure**	**Dosage and method**	**Frequency/duration**	**Purpose/rationale**
Internal medications	Ashwagandha tablets	2 tablets with *Punarnavadi Kashaya*	Twice daily	*Vata-shamana*, adaptogenic, neurotonic
	Punarnavadi Kashaya	15 mL decoction + 45 mL warm water	Twice daily before food	Reduces *Shopha* (edema), improves microcirculation
	Tiktaka Ghrita	10 mL after early dinner	Once daily	*Pitta-Vata* pacifying, *Rasayana* for ocular tissue
	Kalyanaka Ghrita	10 mL after early dinner (with *Tiktaka Ghrita*)	Once daily	*Medhya Rasayana*, supports ocular and mental health
	Triphala Churna	10 g mixed with 1 tsp *Ghrita* and $\frac{1}{2}$ tsp *Madhu* (honey)	At bedtime	*Netra balya*, detoxification, antioxidant
External/local therapies	Siro Pichu	5 mL *Brahmi Ghrita* retained on scalp vertex	Once daily	Calms *Vata*, nourishes cranial and ocular tissues
	Pratimarśa Nasya	5 drops of *Anu Taila* instilled in each nostril	Twice daily	*Vata-shamana* in head region, enhances ocular perfusion
	Añjana Karma	*Elaneer Kozhumbu* applied to lower conjunctival sac	Once daily	*Netra śodhana* and *balya* (cleansing and strengthening)
Lifestyle modifications	–	• Regular and timely sleep • Timely meals • 20 min guided meditation daily • Regulated screen exposure	Continuous	Prevents *Vata aggravation*, supports ocular and mental stability

**Table 2:** Summary of outcomes.

**Case**	**Age/sex**	**Eye affected**	**Prakriti**	**CMT (μm) baseline**	**CMT (μm) post-Tx**	**Baseline VA**	**Final VA**	**Duration of therapy**	**Recurrence**
1	65 /M	Left	Vata	488	Normal-231	6/36	6/9	4 months	No
2	40 /F	Left	Vata	457	Normal-243	6/60	6/6	6 months	No
3	46 /M	Left	Vata	339	Normal-227	6/12	6/6	15 months	No

## 3 Discussion

### 3.1 Limitations and future scope

As with any case series, the observations presented here are based on a small number of patients and are best interpreted as preliminary insights rather than definitive trends. The uniform Prakriti profile of the participants and the individualized duration of treatment reflect the realities of routine clinical practice, but they naturally limit broader generalization. The Ayurvedic intervention was delivered as an integrated therapeutic package, which is characteristic of the system's holistic framework; however, this makes it difficult to distinguish the contribution of individual components. These initial findings, while modest in scale, open avenues for more structured, prospective studies that include diverse Prakriti types, standardized symptom assessments, and controlled comparison groups to deepen understanding of constitution-based responses in CSR.

The current case series presents three patients of Central Serous Retinopathy (CSR), all identified as having a Vata Prakriti, who responded favorably to a uniform Ayurvedic management protocol. The consistent anatomical and visual improvement observed across these cases invites a deeper look into the relationship between Prakriti, disease susceptibility, and therapeutic responsiveness in ocular pathology. The Prakriti of all three patients was assessed using the standardized CCRAS Prakriti Assessment Questionnaire, a validated tool widely used in Ayurvedic clinical research. This questionnaire evaluates physical, physiological, and psychological attributes to derive an objective Prakriti score, reducing subjective bias in constitution classification. Its use in this study strengthens the reliability of identifying Vata predominance and supports the interpretation of Prakriti-linked susceptibility in CSR.

#### 3.1.1 Parallels between Type A personality and Vata Prakriti

Although CSR has long been associated with Type A personality traits, the behavioral and physiological overlap with Vata Prakriti provides a new interpretive framework. Type A individuals tend to be mentally agile, impatient, and emotionally reactive, exhibiting a pattern of sympathetic hyperarousal and reduced parasympathetic modulation. Similarly, classical Ayurvedic texts describe Vata dominant persons as alert, active, and quickly responsive but also prone to anxiety, irregular sleep, and overexertion.

These shared characteristics suggest that Vata Prakriti may correspond to a constitution with heightened neural responsiveness and variable autonomic tone. From a psychophysiological standpoint, both patterns reflect an imbalance between activity and regulation- a dynamic instability that may underlie the stress-sensitive choroidal dysfunction seen in CSR. The present cases, all in Vata individuals, thus align with the modern understanding that CSR tends to occur in persons with higher adrenergic reactivity and stress sensitivity.

#### 3.1.2 Nature of Shopha and its correspondence with CSR

In Ayurvedic nosology, retinal edema or fluid accumulation can be interpreted as a localized form of Shopha (swelling or inflammatory accumulation). Among the three types of Shopha-Vataja, Pittaja, and Kaphaja, the Vataja variety is described as *kshiprothana shamah* (rapid to onset and subside), *punarbhavi* (recurrent in nature), and *ruksha-khara* (associated with dryness and degeneration) (8).

These attributes resonate closely with the clinical course of CSR, which often manifests abruptly following stress and is notorious for its recurrent episodes even after apparent resolution. The sudden accumulation and spontaneous fluctuation of subretinal fluid, the absence of marked inflammatory signs, and the tendency toward chronicity can all be viewed as expressions of Vata-dominant pathology at the ocular level-a Vataja Shopha of the macular region. The rapid onset and recurrence observed in CSR, therefore, not only parallel the classical descriptions of Vataja Shopha but also explain why patients with Vata Prakriti, by nature predisposed to Vata disorders, may be more vulnerable to such retinal fluid dynamics.

#### 3.1.3 Predisposition of Vata Prakriti to ocular disorders and the therapeutic approach

Individuals of Vata Prakriti are known to be more susceptible to diseases characterized by instability, degeneration, and irregular movement, pathophysiological domains governed by Vata Dosha (9). Ocular tissues, being delicate and rich in neural and vascular structures, are particularly sensitive to such derangements. When Vata aggravation localizes in the *Netra*, it manifests as *Vataja Netra Roga*, which includes symptoms like *Vakram rjuvapi manyate* (visual distortion), *Aaviladarshana* (blurred vision), and fluctuating clarity of vision, remarkably similar to the subjective complaints in CSR (10).

The Ayurvedic management employed in these cases primarily involved Shamana therapy (pacification of the aggravated Dosha) and Rasayana therapy (restorative and rejuvenative support). Formulations such as *Ashwagandha* and *Kalyanaka Ghrita* are well-recognized for their adaptogenic and neuroprotective effects, which may help modulate stress-related sympathetic overactivity (11). *Punarnavadi Kashaya* and *Tiktaka Ghrita* act as anti-edematous and detoxifying agents, addressing the fluid imbalance represented by the macular edema. Local therapies like *Shiro Pichu* with *Brahmi Ghrita* and *Pratimarsha Nasya* with *Anu Taila* contribute to the stabilization of Vata in the head region, supporting ocular nourishment and functional equilibrium.

The addition of *Triphala Churna* with ghee and honey serves both as a Rasayana and ocular tonic, reinforcing the regenerative aspect of therapy. The emphasis on lifestyle correction- timely sleep, regulated diet, meditation, and reduced visual strain, further aligns with Vata pacification principles.

From a clinical integration standpoint, the convergence of Ayurvedic and biomedical perspectives becomes apparent; while modern research highlights the role of sympathetic overdrive and stress hormones in CSR, Ayurveda attributes similar dynamics to Vata aggravation. The successful outcomes in this series underscore that targeted pacification and rejuvenation of Vata through Shamana and Rasayana therapies may restore both structural and functional homeostasis of the retina.

In keeping with authentic Ayurvedic practice, the therapeutic outcome in these cases reflects the integrated effect of internal medicines, external ocular therapies, lifestyle regulation, and dietary guidance, which function synergistically and cannot be meaningfully separated within this study design.

While all three patients in this series were identified as Vata-predominant, this small and homogenous sample does not allow any inference of association between Prakriti type and CSR. Instead, this observation should be viewed as preliminary and hypothesis-generating, underscoring the need for future prospective studies involving CSR patients with diverse Prakriti profiles to explore whether such a relationship truly exists or if the present pattern reflects selection bias.

## 4 Conclusion

The outcomes from this case series highlight a noteworthy association between Central Serous Retinopathy (CSR) and Vata Prakriti, suggesting that constitutional predisposition may play a key role in disease expression and progression. The favorable anatomical and functional recovery observed in all three patients underscores the value of personalized, Prakriti-based Ayurvedic interventions, particularly therapies directed at pacifying Vata and enhancing ocular resilience through Rasayana. Integrating such individualized approaches alongside modern ophthalmic understanding could open new avenues for precision eye care, offering better visual outcomes and reduced recurrence in CSR management.

## Data Availability

The original contributions presented in the study are included in the article/supplementary material, further inquiries can be directed to the corresponding author/s.
